# The Recent Advancement in Unmanned Aerial Vehicle Tracking Antenna: A Review

**DOI:** 10.3390/s21165662

**Published:** 2021-08-23

**Authors:** Anabi Hilary Kelechi, Mohammed H. Alsharif, Damilare Abdulbasit Oluwole, Philip Achimugu, Osichinaka Ubadike, Jamel Nebhen, Atayero Aaron-Anthony, Peerapong Uthansakul

**Affiliations:** 1 Department of Aerospace Engineering, Faculty of Air Engineering, AirForce Institute of Technology, Kaduna 800282, Nigeria; hk.anabi@afit.edu.ng (A.H.K.); damilareoluwole@afit.edu.ng (D.A.O.); ocubadike@afit.edu.ng (O.U.); 2 Department of Electrical Engineering, College of Electronics and Information Engineering, Sejong University, 209 Neungdong-ro, Gwangjin-gu, Seoul 05006, Korea; 3 Department of Computer Science, Faculty of Computing, AirForce Institute of Technology, Kaduna 800282, Nigeria; p.achimugu@afit.edu.ng; 4 College of Computer Engineering and Sciences, Prince Sattam bin Abdulaziz University, P.O. Box 151, Alkharj 11942, Saudi Arabia; j.nebhn@psau.edu.sa; 5 Department of Electrical and Information Engineering, Covenant University, Ota 112233, Ogun State, Nigeria; atayero@covenantuniversity.edu.ng; 6 School of Telecommunication Engineering, Suranaree University of Technology, Nakhon Ratchasima 30000, Thailand

**Keywords:** UAV, tracking antenna system, azimuth angle, elevation angle, range, MIMO technology, beam steering, antenna array

## Abstract

Unmanned aerial vehicle (UAV) antenna tracking system is an electromechanical component designed to track and steer the signal beams from the ground control station (GCS) to the airborne platform for optimum signal alignment. In a tracking system, an antenna continuously tracks a moving target and records their position. A UAV tracking antenna system is susceptible to signal loss if omnidirectional antenna is deployed as the preferred design. Therefore, to achieve longer UAV distance communication, there is a need for directional high gain antenna. From design principle, directional antennas are known to focus their signal energy in a particular direction viewed from their radiation pattern which is concentrated in a particular azimuth direction. Unfortunately, a directional antenna is limited by angle, thus, it must always be directed to the target. The other limitation of a UAV mechanical beam steering system is that the system is expensive to maintain and with low reliability. To solve this problem, we are proposing the use of MIMO technology as a readily available technology for UAV beyond line of sight technology. Although UAV antenna tracking is domiciled in the mechanical beam steering arrangement, this study shows that this native technology could be usurped by MIMO beam forming.

## 1. Introduction

**Figure 1:**
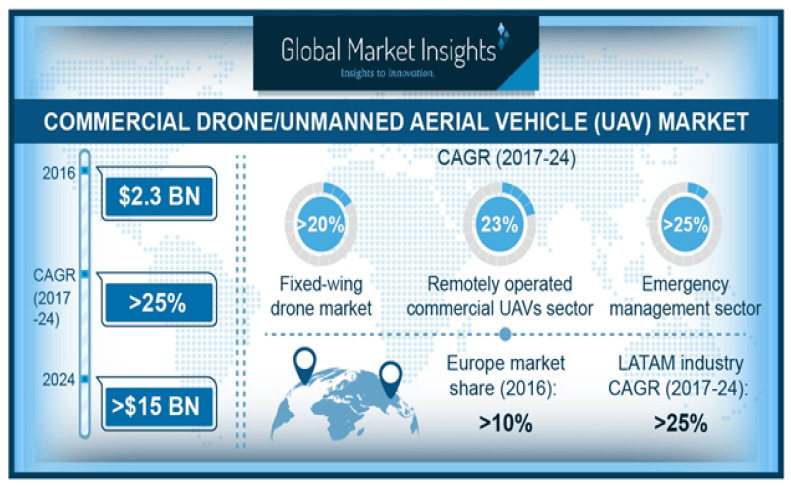
UAV market outlook [13].

During the last two decades, unmanned aerial vehicle (UAV) has been deployed to perform high risky operations where deploying humans presents great danger to their health notably in fire-fighting [1,2], chemical spraying [3,4,5], search and rescue missions [5,6], disaster network communication [7,8] and surveillance operations [9]. Undoubtedly, UAV will be pivotal in medical and blood delivery for troops in the frontline when it is dangerous to send in medical personnel. The market of UAV has a positive outlook, as could be seen from Figure 1. The major drivers of the UAV market are the remotely operated commercial UAV and the emergency management sector. Particular interest is the remotely operated commercial UAV where the channel model ranges from direct line of sight (LoS) to non-LoS propagation communication [10]. UAVs are deployed in urban, semi-rural, and rural environments which are fully captured using LoS and non-LoS scenario. Each environment characterized by different link obstacle topology. For instance, in the rural area where there is less obstacle, the channel model conforms to the free space path loss model suitable for LoS communication. Different from the NLoS model, the UAV ground control station (GCS) deploys the LoS model to focus the antenna beam directly to the airborne UAV using a highly directional antenna rather than an omni-directional antenna to maximize the received signal power.

Furthermore, the assigned UAV transmit powers which varies might become inadequate as communication range increases. For instance, wifi operated UAV is limited to 20 dBm while the LTE operated UAV is limited to 24 dBm in uplink and 43–48 dBm in downlink) [10]. Hence, there is a need to rely on tracking antenna to deliver the needed signals over a wider communication range in unpredictable wireless communications link. Obviously, using highly directional antenna enhances the received signal strength (RSS) of the airborne platform. Thus, there is a need to reduce co-channel and cross channel interferences. Additionally, the need for automatic repeat request (ARQ) as well as hybrid automatic repeat request (HARQ) in the face of loss of signal when deployed in autonomous fashion are also reduced. Moreover, the aerial platform records a higher signal-to-interference-noise ratio (SINR) when a packet is successfully delivered. From the perspective of energy efficiency and network lifetime, deploying omni-directional antenna connotes the use of more energy to achieve a targeted reliable communication link between the GCS and the air-borne platforms. Intuitively, directional antenna is highly desirable for UAV communication because it improves pointing accuracy, and minimizes the antenna sidelobes and antenna beamwidth. In high-frequency band communication, sustaining precision communication between the ground station and the airborne platform requires significant improved pointing accuracy because of the short wavelength and range [11,12].

Meanwhile, using a directional antenna is limited by azimuth and elevation angles, and must always be directed to the target [14]. It is important to note that optimal directional antenna should consider UAV look angles (yaw, pitch, and roll) which are dynamic as they are motion-based. Therefore, the UAV tracking antenna must overcome this limitation by constantly adjusting the beam target direction. Keeping this in mind, different researchers have explored several ways to address this problem and have discussed in this article. The major contributions of this work are listed below:

- This work provides an exhaustive review on the state-of-the-art UAV antenna tracking technology as well some discussion on tracking antenna fundamentals.
- Instead of relying on the electro-mechanical UAV antenna tracking technology, this work proposes the adoption of MIMO beam-forming technology as an alternative to the electro-mechanical UAV antenna tracking.
- Lastly, we propose the use of sectorized beam-forming for UAV antenna tracking which is capable of providing 0–360° coverage.

This work is limited to UAV ground and air communication link leaving behind the UAV satellite link. The rest of the paper is organized as follows. Section 2 presents an overview of a UAV antenna tracking system. Section 3, provides discussions on the various types of antenna suitable to be deployed for a UAV antenna tracking system. Section 4, performs a state-of-the-art on a UAV antenna tracking system, analyzing the recent trends in the field. Section 5 is dedicated to MIMO technology as a suitable replacement to the mechanical UAV antenna tracking. The paper is concluded in Section 6.

## 2. UAV Tracking System Overview

**Figure 2:**
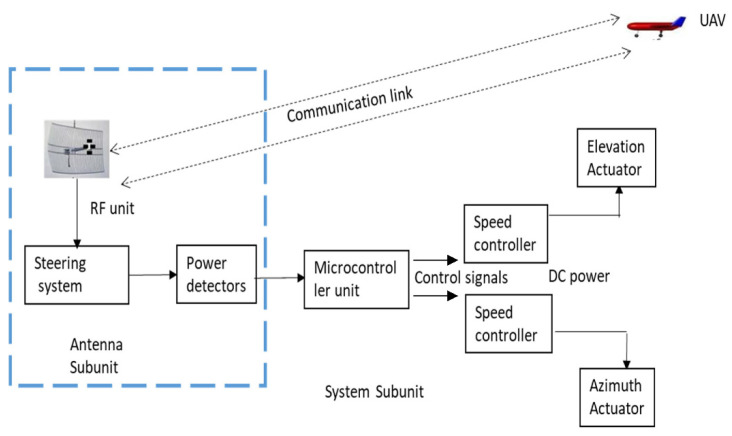
A UAV tracking antenna system.

A UAV tracking antenna system as depicted in Figure 2 which consists of the antenna subunit and the system subunit. The antenna subunit comprises of the antenna, the steering block, motors, gears, communication link, and power detectors. Meanwhile, the system subunit comprises of elevation, azimuth, the microprocessor, and signal detection system.

### 2.1. Communication Link

Communication link uses a radio-frequency (RF) medium to transmit and receive information packet to and from the UAV. There are various frequencies currently deployed. The deployed frequencies are based on UAV brand as well as functionality of the UAV. As seen in certain UAVs, frequencies, such as 433 MHz and 915 MHz, are used for telemetry, while 1.2 GHz and 2.4 GHz are used for video transmission [10,15]. The 433 MHz and 915 MHz are used because of their longer transmitting range capability. However, they suffer from low bandwidth and, hence, are not ideal to transmit video signals. The major disadvantage of using 1.2 GHz and 2.4 GHz is that they are simply designed for short range.

### 2.2. Steering System

The steering system is significant in the overall functionality of the UAV tracking system. The primary task of this unit is to rotate the antenna in azimuth and elevation look angles. At present, there are two types of UAV steering systems, namely (i) mechanical system; and (ii) electrical (or electronic) steering.

***(a)***  ***The mechanical steering system***

The mechanical steering system is the most energy-consuming unit of tracking system because it needs energy to drive the motors and overcome the torque [16].The energy loss is seen in the form of heat energy which are dissipated in the metallic enclosures. There are several types of motors in use for the UAV tracking system, namely (i) stepper motors [17,18]; (ii) DC motors [19,20]; (iii) geared DC motors [21,22]; (iv) brushless DC (BLDC) motors [23,24]; and (v) brushless AC (BLAC) motors [25]. It has been reported that the steeping motor is the most effective in designing steering antenna [26]. Whereas, DC motors are characterized as having low life and low heating of the armature windings. However, they are very cheap. Furthermore, the mechanical steering operational principle is based on closed-loop and open-loop mechanisms. In a closed-loop mechanism, there is a feedback system to control the motors while open-loop depicts the absence of feedback mechanism effectively there is no control system. Consequently, the tracking unit control relies on positioning sensors, such as gyroscope and inertial measurement unit (IMU), for guidance [27]. This type of control mechanism is suitable for a land to mobile satellite services and it is not affected by shadowing and blocking signal components.

***(b)***  ***The electrical steering system***

Instead of utilizing the mechanical type of beam-steering mechanism, electrical beam steering can be utilized. In electrical beam steering, phase shifters add propagation delay into the system to induce a phase shift [28]. Phase shifters can be analog or digital, active or passive. RF micro-electrical mechanical systems (MEMs) phase shifters are available for passive high-performance antenna arrays for Ka-band (26–40 GHz) applications that exhibit superior performance to solid-state alternatives. Analog phase shifters are controlled by a voltage compared to a digital phase shift which is specified with a bit precision related to a phase shift at a frequency or over a frequency band. The electrical steering system has more considerable tracking speed, acceleration, and mean time between failure (MTBF) over its mechanical counterpart. Buoyed by their compact structure, they are more suitable to be installed in mini structures and have lower production losses [29]. However, they are less efficient and have higher losses than their mechanical counterpart. Some other techniques to achieve electronic/electrical beam steering include active phased arrays, passive phased arrays, and switched multiple-beam antennas [30].

### 2.3. Microcontroller Unit

This is the “brainbox” of the UAV tracking system as they fetch and execute instructions through programmable memory. Microcontrollers and embedded controllers come in various bit configuration, such as 4, 8, 32, 64, and 128 bits. The 32-bits microcontroller is primarily designed for automatic control of devices and was recently used in the design of a UAV tracking system [31]. There are different microcontroller types, namely, 8051, PIC, AVR, ARM, Renesas, MSP, Hitachi, and Motorola [32,33]. The Renesas microcontroller offers attractive features, such as low power, high performance, modest packages, and the largest range of memory sizes combined together with character-rich peripherals [34,35]. Apart from the fuzzy logic controller (FLC), the three most popular control algorithms are (i) proportional–integral (PI), (ii) linear quadratic gaussian (LQG), and (iii) H∞ UAV. A study on the performance, precision, tracking attributes, and shortcomings has been evaluated [36]. Reference [37] designed, discussed, and implemented a control system consisting of a BLDC motor and a PI controller. Expectedly, the PI is responsible for reducing position error and improving response towards the antennas. Although the PI is the simplest and most reliable algorithm, it suffers from a lack of stringent pointing accuracy and, hence, cannot be used to control large antennas. This limitation can be overcome by the use of a LQG algorithm. LQG is equipped with a shorter response time and turbulence rejection attributes. The LQG can be further enhanced by the use of H∞. The H∞ algorithm is noted for acceleration limits because of the existing motor drives and gear system. This limitation can be addressed by the use of command pre-processor in software that modifies antenna commands. Reference [38] implemented an antenna control system using a step algorithm method around H∞ control algorithm. Results indicate that the algorithm was able to overcome significant torque disturbances from wind pressures and gusts on the antenna structures, as well as bearing and aerodynamic frictions. Reference [39] presented an overview on the development and application of antenna control systems, while [40] presented performance analysis of PID and LQG control algorithms for antenna position control system.

## 3. Antenna Overview

**Figure 3:**
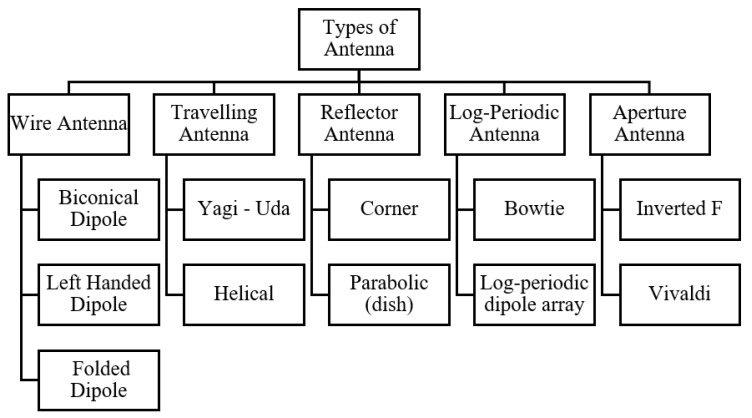
The major types of antenna discussed.

Antennas deployed in a UAV tracking system must conform to either as a steering beam (SB) or an array antenna (AA) format. The SB configuration involves just only one antenna unit, such as parabolic dish. However, the AA entails the fusion of many micro antenna elements to functions as an antenna structure. In this section, we present a brief overview on the different antenna and antenna arrays suitable for deployment as a UAV antenna tracking system. Some of the examples of the notable antennas are classified, as shown in Figure 3 and discussed hereafter.

### 3.1. Wire Antenna

Wire antenna is a radio antenna with a long wire suspended above the ground. Although ground terminal is very crucial in antenna transmission range, in this specific design, the ground plays a dominant role. The length of the wire has no effect on the wavelength of the radio used, but for convenience instead [41,42]. The wire can be straight or strung forth and back between walls and trees. This type of antenna is not as effective as those antennas whose length are adjustable to resonate to the wavelength intended.

### 3.2. Biconical Dipole Antenna

**Figure 4:**
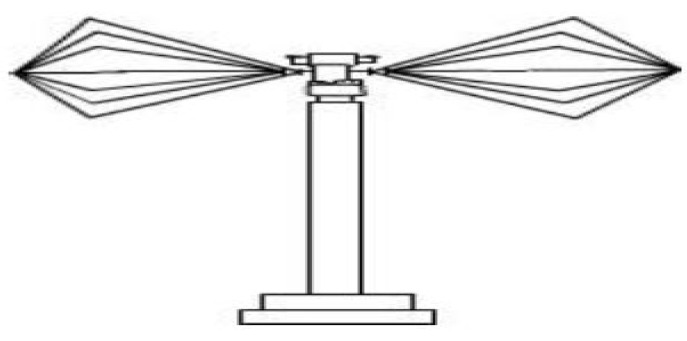
Biconical antenna.

Biconical antennas (also called butterfly or bowtie antennas) are broadband dipole antennas made up of two roughly conical conductive objects. These antennas have char-acteristics like that of dipole, with a larger bandwidth achieved as a result of the double cone elements structure. The application of biconical antennas are evident in emission testing, immunity testing, and measurement of shielding effectiveness in anechoic chamber [43,44]. Figure 4 shows a typical biconical antenna.

### 3.3. Left Handed Dipole Antenna

The left-handed dipole antenna is a new kind of antenna. Its name derives from its left-handed mode of transmission [45,46]. The design of this antenna is fundamentally built on shunt inductors and capacitors. The capacitor is placed by the line that prompts current of different amplitude on either side. This current does not totally cross out in the far-field, thus, it transmits.

### 3.4. Folded Dipole Antenna

The folded dipole antenna is one of the simplest and cheapest antenna extensively deployed in wireless transmission. It is compact and can be easily manufactured and installed. The design is based on the wires folded but with open ends. The folded dipole antennas are wide loop in nature. The radiation pattern of a folded dipole antenna is the same as that of a dipole antenna [47,48,49]. These antennas attributes help the antenna to gain traction, as dipole antennas are known to have high directivity and high gain.

### 3.5. Travelling Antenna

The primary radiating mechanism of the travelling antenna is based on a travelling wave in the form of a guiding structure. A travelling antenna is different from others because the radio waves are generated by the radio frequency current in a single direction [50,51]. They are non-resonant and, as such, their bandwidth is wider than their resonant antennas counterpart [52]. Some of the examples of a travelling antenna are explained below.

#### 3.5.1. Yagi–Uda Antenna

**Figure 5:**
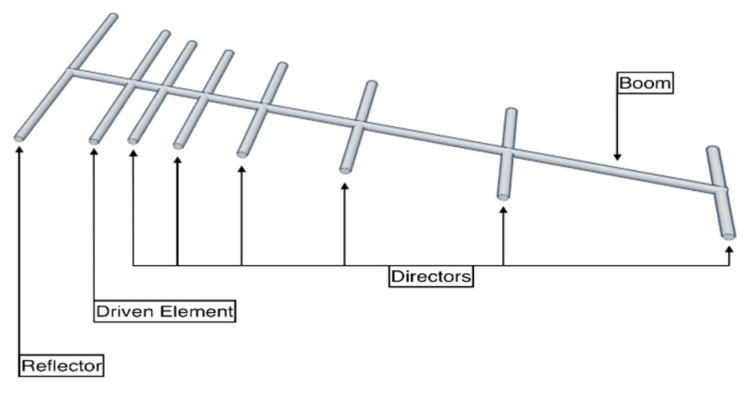
Yagi–Uda antenna [55].

The Yagi–Uda antenna, also known as Yagi antenna or Yagi, has guided-course arrangement, with dipole and some extra parasitic components firmly coupled together, known as the reflector and directors. The main components of a Yagi antenna are the reflectors, directors, and dipole. The Yagi antenna has seen wide application in many areas, such as UHF/VHF radars, wind profiler systems, and phased Doppler radars. Its performance strongly depends on reflector, dipole, feeder, and director [53,54]. Figure 5 demonstrates the structure of the Yagi–Uda antenna [55].

#### 3.5.2. Helical Antenna

**Figure 6:**
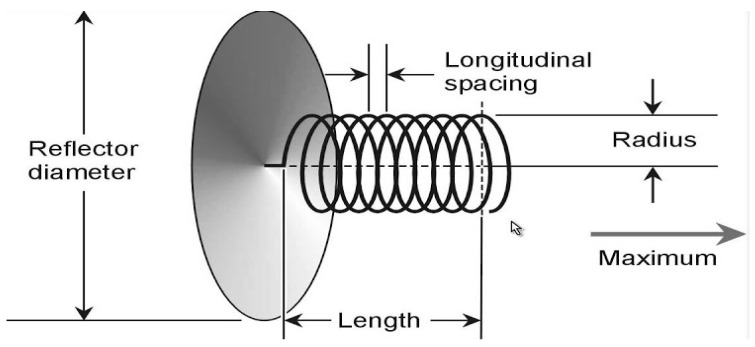
Helical antenna [59].

A helical antenna has one or more conducting wires wound in such a way that the shape looks like a helix and hence the name. Helical antenna has been extensively used in practice for many years because of the practical emission and how easy it is to use [56,57]. The properties of this type of antenna is extraordinary and exceptional and as such, they are widely used in getting microwaves from VHF [58]. The helical antenna is also used in satellite communication due to the high gain requirement. Figure 6 demonstrates the structure of the helical antenna [59].

### 3.6. Reflector Antenna

The reflector antenna simply modifies the radiation pattern of an antenna by redirecting the received electromagnetic energy in the range of the electromagnetic spectrum wavelength, thereby increasing the gain in a particular direction.

#### 3.6.1. Corner Reflector Antenna

**Figure 7:**
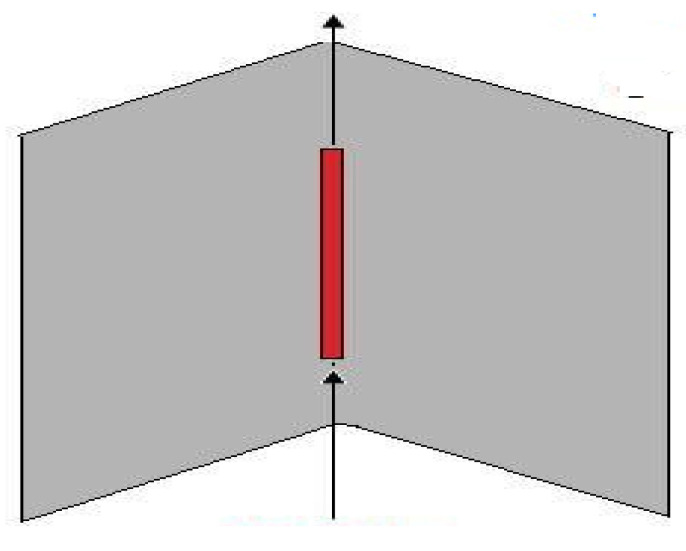
Corner reflector antenna [60].

The corner reflector type of antenna is very effective yet very simple. The antenna shown in Figure 7 has plane reflectors in the form of a panel and a dipole element [60].

The antenna arrangement prohibits radiation in backwards and sideways direction and, as such, they are more directional [61,62]. A prototype of reconfigurable corner reflector using PIN-diode-switched frequency selective surfaces (FSS) has been developed [63]. A reflector aperture angle of 45° is prototyped for application at 4 GHz, and its pattern characteristics are altered by on-off state biasing of the PIN diodes. Readers interested in the theory of corner reflectors with tilted dipole can consult [64].

#### 3.6.2. Parabolic Reflector (Dish Antenna)

The parabolic reflector antenna has two types. The first is the right cylinder while the second one is paraboloid. The cylinder type uses linear array, linear dipole, and slotted wavelength to feed. The paraboloid, on the other hand, uses pyramidal or paraboloid conical to feed. The radiation pattern of this antenna is strongly determined by the radiation pattern of the feeder used as well as the reflector measurements and material [65,66]. An evaluation study on the desirability of defocusing a large-sized multi-beam hybrid parabolic antenna (MBHPA) has been performed [67]. Defocusing means displacement of the AA from the focus, in the interests of rarefaction of the AA. Results indicate that the idea of defocusing, which is tempting at first glance, does not benefit in the gain factor of the beams in comparison with un-displaced AA with the same spacing of the array elements.

### 3.7. Log-Periodic Antenna

A log-periodic antenna (also known as a log-periodic aerial or array) is a narrow-beam, directional, and multi-element antenna with a broad bandwidth. This type of antenna has the capability to operate over a wide range of frequencies. The log-periodic antenna supports a wide band of frequencies and, as a result of that, it is usually used in television antenna that specifically work in the VHF band. The log-periodic antenna is structurally similar to the yagi antenna, but with a distinct design. The log-periodic antenna comes in various formats as discussed herein.

#### 3.7.1. Bowtie Antenna

**Figure 8:**
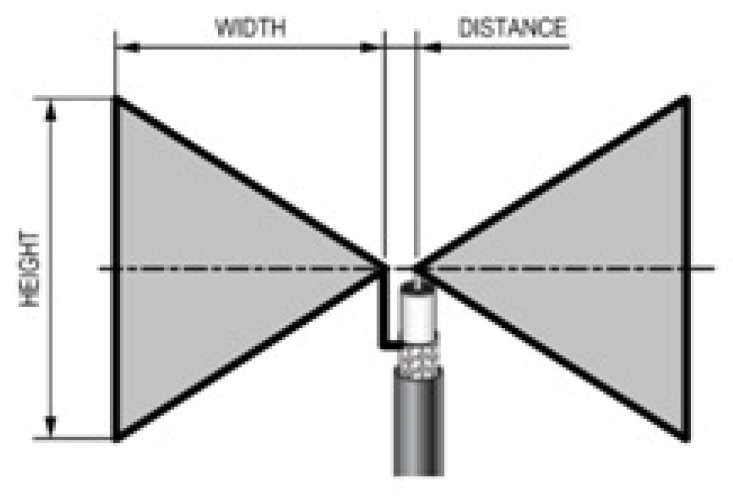
Bowtie antenna [60].

The demand for low-cost, effective, and less-weight antennas in the communication network and multi-band application has brought about the design of this antenna. Recently, communication network requires light, easy working, and moveable antennas. Due to its structure, it is known as a butterfly antenna. There are different types of bowtie antenna: the bowtie slot antenna, the double-sided triangular bowtie antenna, the bowtie antenna microstrip fed, the slotted bowtie patch antenna, and the CPW fed curved bow tie slot antenna. The bow tie antenna has two mirrors placed on a rectangular patch. The output of this design is limited by distance and the alignment of the mirrors. This type of antennas are widely used in mobile communication networks and wireless systems [68,69]. Figure 8 demonstrates the bowtie antenna.

#### 3.7.2. Log-Periodic Dipole Array Antenna

**Figure 9:**
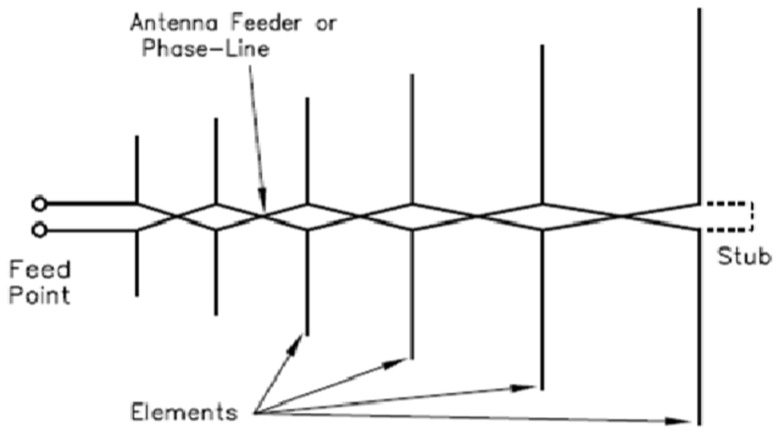
Log-periodic dipole array antenna [72].

This antenna is always very handy in cases where there are wideband applications. It operates in a VHF range from 30 MHz to 300 MHz. The bandwidth of the antenna is usually greater than 10:1, and they are referred to as independent antenna. The features of this antenna, such as gain, input impedance, and radiation, changes periodically with respect to the logarithm of the frequency space. This is referred to as a log-periodic array [70,71]. Figure 9 demonstrates the structure of the log-periodic dipole array antenna.

### 3.8. Aperture Antenna

Aperture antennas are one of the most popular type of antennas; they emit electromagnetic waves through an opening also referred to as an aperture. The transmitted and received electromagnetic waves are obtained from waveguide into space. The directivity of this type of antenna is low and, as such, they are preferred design in frequencies, such as microwave frequency and UHF. Some examples are:

#### 3.8.1. Inverted F Antenna

The appetite for an inverted F antenna surged with an increased demand for light-weight and simple yet effective antennas in many commercial modern communication networks. The Bluetooth technology uses the inverted-F antenna. The pattern of radiation of the inverted-F antenna is omnidirectional in nature with a bandwidth of 250 MHz. Another good use of this type of antenna is its effectiveness for indoor communication [73,74,75]. The inverted-F arrays can be easily fabricated using microstrip materials in which conductors usually copper can be etched out using a suitable machine. Since the antenna is domiciled in the omnidirectional domain, it may not be suitable for highly directional UAV tracking antennas with long-range communication.

#### 3.8.2. Vivaldi Antenna

**Figure 10:**
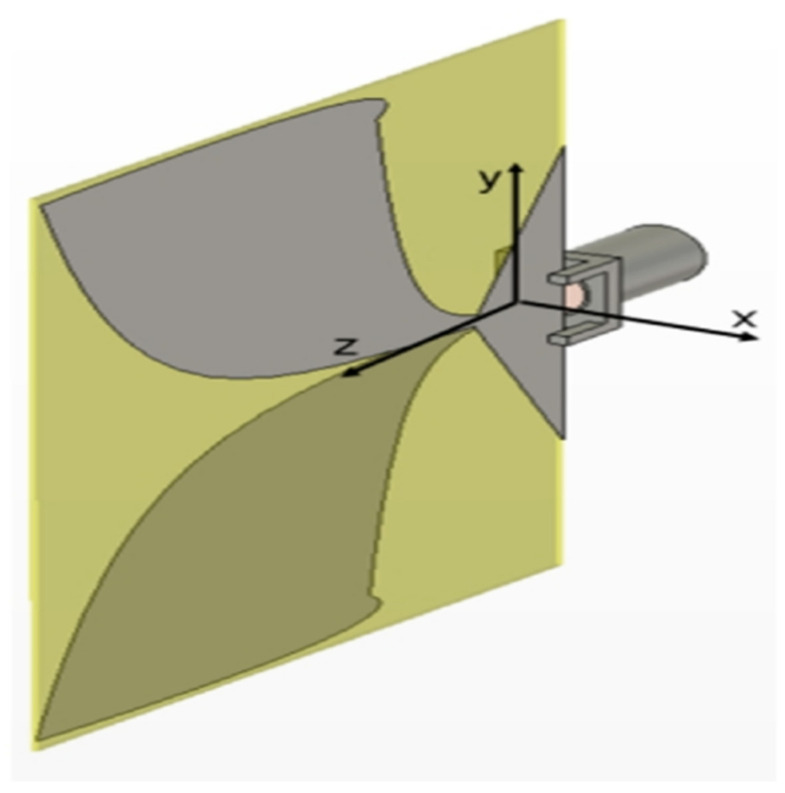
Vivaldi antenna [81].

The low cost, simplicity, and high band operation of Vivaldi antenna has brought about its wide application and usage in many devices [76]. These antennas are used in radar application and are also used in microwave imaging [77]. The antenna has received attention as a suitable candidate for emerging technologies, such as 5G [78], wideband communication using image theory technique [79] and high-gain antenna using optical lens [80]. Figure 10 illustrate a design example of Vivaldi antenna [81].

### 3.9. Array Antenna Model

Imagine an arbitrary array antenna consisting of several radiating elements *M* separated by a distance *d.* Arrays are primarily used as directors to align the incident and reflected signals to a desired beamwidth. Thus, the array factor (AF) of the incoming signal is modelled as [82,83]:

(1) $$\mathrm{AF}\left(\theta \right)=1+e^{j\Phi }+e^{2j\Phi }+\cdots +e^{\left(M-1\right)j\Phi }$$

where array phase function  (Φ)=kdsin(θ)+β and the propagation constant (k)=2π/λ, λ denotes the wavelength, β is the phase angle between the elements, θ is the desired angle of arrival (AoA) of the incoming signal. The propagation constant is a measure of changes in a sinusoidal electromagnetic wave in terms of amplitude and phase, while propagating through a medium. β denotes a control parameter which steers the antenna between the broad side and the end-fire. In other words, it is responsible for cancelling out the interference, resulting from the various antenna elements. When β = 0°, then θ = 90°. This configuration is known as the end-fire condition and the received signal is maximum. On the contrary, when β = 180°, then θ = 180°, then broadside occurs, signifying the worst received signal strength. Increasing the array elements results in high directivity, high gain, and low beam width, as shown in Table 1. Thus, having a higher number of array elements, as observed in Yagi, log-periodic, helical antenna results in high gain. The close format of AF is stated as [84]:

(2) AF(θ)=sin(MΦ2)sin(Φ2)

Hence, the directivity of such an array is equal to *M*. It is plausible to derive the beam width of an arbitrary *M* isotropic elements spaced by a wavelength using this formula [83]:

(3) $$BW=\frac{51\lambda }{Md}$$

## 4. Recent Advancement on UAV Tracking Antenna

In this section, we conduct a survey on the recent trends in the area of UAV tracking antenna. Furthermore, the fundamentals of UAV tracking antenna technology are also analyzed.

### 4.1. UAV Tracking Positioning Parameters

The essence of the UAV tracking antenna is to align the signals from the GCS according to the orientation of UAV yaw, pitch, and roll during flight mission. Taking into consideration the position of the celestial bodies. These parameters are important in the absence of or loss of GPS signal. The three notable UAV tracking parameters are: (i) azimuth angle; (ii) elevation angle; and (iii) range, which are all briefly discussed.

**Figure 11:**
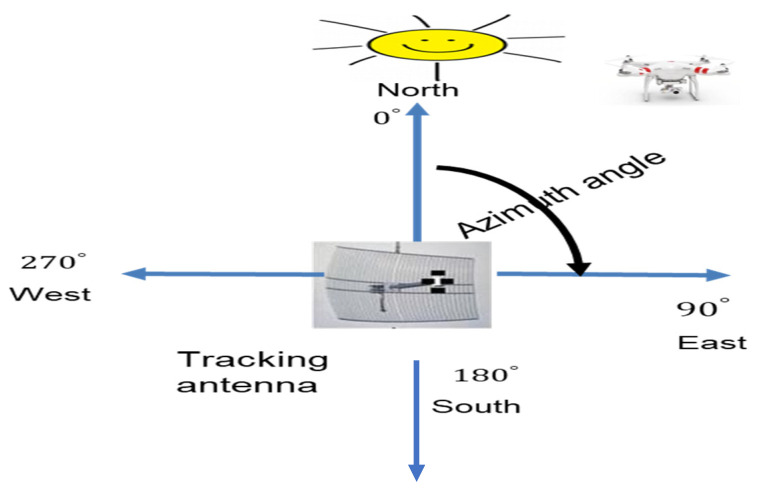
Azimuth angle.

The azimuth is the angle between North, measured clockwise referenced by any of the celestial bodies in most cases, and the sun. In the absence of GPS, the azimuth can be obtained using the compass bearing system. The azimuth is deployed to measure the angle ranging from 0–360° [16,85]. Figure 11 illustrates the azimuth angle. The azimuth generally depicts the antenna directivity and, by extension, the antenna gain. An antenna with a uniform azimuth exhibits omnidirectional property while an antenna with partial azimuth is considered as highly directional.

The elevation angle herein refers to the vertical angle distance between the UAV tracking antenna local horizon or, also called, the observer’s local plane and the UAV. The angle of elevation can be calculated by knowing the UAV altitude and the horizontal distance. The range is simply the distance along a great circle between the UAV and the tracking system. Great circle is the shortest distance along the earth equator. In a clear line of sight scenario, the range can be obtained by using a Friis transmission model, ignoring the earth bulge factor [83]. Alternatively, spherical law of cosines equations can be used [31].

### 4.2. Tracking Orientation

Tracking orientation is responsible for obtaining the angular displacement of a body as well the coordinates. For the angular displacement of a moving UAV which comprises of yaw ψ, pitch *θ*, and roll ϕ to be properly tracked, two popular strategies are often deployed which are (i) keeping the coordinates constant; and (ii) allowing the object in this case the airborne platform to rotate. The other approaches are discussed below.

#### 4.2.1. Ground-to-Air Tracking Antenna

Ground-to-air antenna tracking systems relies on the determination, measurement, and comparison of signal strength gradient from the GCS to the airborne platform. This scheme motivates the search for the optimal elevation and azimuth angle between the two antennas involved [86,87]. The ground-to-air tracking antenna system is a very simple and efficient approach of tracking a target without knowledge of the air vehicle attitudes. The use of a circularly polarized microstrip antenna array to increase the bandwidth in S-band for UAV ground to air transmission has been explored [88]. The simulation results of the array element showed that its relative bandwidth is 21.7% with gain greater than 5 dBi and VSWR ≤ 1.25. Similarly, reference [31] built an antenna tracker using GPS and a 32-bit microcontroller, which is of two degrees of freedom. The system took into consideration the altitude of the vehicle and was able to provide an azimuth angle of 360 degrees and a pitch angle of 90 degrees. This approach will be ideal for a UAV case, considering the fact that airborne platform is constrained by weight, size and power (swap).

#### 4.2.2. Air-to-Ground Tracking Antenna

This technique is not as popular as the ground-to-air type of tracking systems. In this design, the tracking is performed by the airborne platform. For the case of the UAV, the UAV uses a directional antenna to send the signal while an omnidirectional antenna is solely for the tracking antenna. The directional antenna on the UAV is desirable because it reduces the burden of detecting the angle of arrival (AoA) of the arrived signal. Despite its challenges, some authors have deployed this scheme [89,90]. Their result shows that the tracking antenna continuously keeps track of and aligns with the base station and GCS, respectively, with the minimum error possible. However, this design is limited to ground based vehicles provided there is an available LoS between the sender and receiver. Reference [91] presented a brief overview of waveforms for UAV air-to-ground communication systems. The researchers broadly divided the modulation schemes into single carrier and multicarrier. The single carrier modulation are: QPSK, APSK, OFDM, MQAM, GMSK, MSK, SOQPSK, SC-FDE. However, the multicarrier is the OFDM. Additionally, [92] conducted air-to-ground UAV tracking although using a MIMO beam-forming technique which showed promising results.

### 4.3. UAV Tracking Techniques

UAV tracking has been implemented based on a variety of techniques, notably using received signal strength indicator (RSSI), using time difference of arrival (TDOA), tracking using global positioning system (GPS), and using monopulse. In this section, we present the latest trend in UAV antenna signal tracking techniques.

#### 4.3.1. UAV Tracking Based on RSSI

Signal strength power measurement, commonly known as the received signal strength indicator (RSSI), is a technique for calculating the distance with respect to signal strength between the UAV and the antenna tracking system [93]. The received signal strength (energy) is measured by the RSSI after quantizing the measured signal energy relative to a position at the receiving end. This RSSI can be used to develop a simple method to estimate location; this is carried out without the addition of extra hardware. The simplest way this can be achieved is to request that the antenna on board the UAV transmit signal. Each node at the receiving end gets a signal. It is then assumed that the UAV is in the direction of the reference node with the highest RSSI [94]. The merits of this type of tracking are [95,96]: (i) low cost; (ii) low battery power energy consumption; (iii) small memory size requirement; and (iv) low processing capabilities. It is obvious that this technique will suffer from UAV poor positioning estimation accuracy as it lacks the potential to perform azimuth, elevation, and polarization calculation. Hence, the UAV rotation and translation are unobservable [97]. In addition, the presence of noise, both white and colored, degrade the positional accuracy of the UAV when using RSSI method [98,99,100]. White noise is a type of noise whose power spectrum is flat at different frequency bands and spreads uniformly. This is in contrast to colored noise whose power spectrum is not flat at different frequency bands and does not spread uniformly. Reference [94] designed a tracking antenna using the principle of RSSI. By using four Yagi antennas, the experiment aim was achieved. The RSSI received by each antenna was detected using four linear technology power detector chip. The result determines the direction the antenna needs to turn to keep track of the UAV. However, although this work did show the use of RSSI, it is not totally accurate. Similarly, [101] proposed RSSI-based heading control for robust long-range aerial communication in UAV networks. The heading control process is mainly divided into two phases, i.e., position estimation and angle adjustment. In the first phase, a proportional-derivative-based tracking controller is designed for each UAV to ensure the consistency of heights, pitch, and roll angles. However, the RSSI functions as an auxiliary measuring component, and then a consensus-based unscented Kalman filtering algorithm is developed to estimate the position of UAVs.

#### 4.3.2. UAV Tracking Based on TDOA

UAV tracking using time difference of arrival (TDOA) involves an estimation of transmitter location using time difference of arrival measurements formed by the correlation of signals received by different nodes at the receiving end [102]. TDOA methods compute the time difference of arrival of a wave at multiple measurement points and calculate the source point direction based on timing and wave comparisons. The basic approach to determining TDOA is to calculate the cross-correlation of the signals arriving at the receiver. The estimate of the TDOA is the delay which helps us maximize the cross-correlation function. Knowing information, such as the location of each receiver and the time of arrival on each, an estimate of the source of the signal can be made. TDOA have generally been applied in different areas due to the following advantages: (i) low cost; (ii) low complexity; and (iii) small in size. However, the performance of TDOA is a function of the signal bandwidth and the performance degrades as the signal bandwidth decreases [103]. In addition, TDOA also require a high-quality time synchronization which is relative to the inverse bandwidth of the signal. Reference [104] focused their research on the performance analysis for TDOA localization using UAVs with flight disturbances, and reference [105] deployed the use of particle filtering for three-dimensional TDoA-based positioning using four anchor nodes driven by statistical models to determine the UAV localization.

#### 4.3.3. UAV Tracking Based on GPS

The significant errors that accompany the previous schemes limits their versatility and, hence, the need for global positioning system (GPS) module. A GPS tracking system is a very common approach in getting vehicles location information in real-time for fleet planning [106]. This mode of tracking uses the unique advantages of the GPS to get the location of the UAV and that of the tracking system. The coordinates (latitude and longitude) of the UAV and tracking antenna are involved, and the various tracking parameters can be estimated. Using models, such as haversine distance and bearing formula, spherical trigonometry, calculation for the azimuth angle, elevation angle, and distance of the UAV from the tracking system, can be calculated. Their advantages resonate around its high precision and accuracy [31,89,107,108]. Many others built an antenna tracker using GPS technology. They were all able to track the UAV and improve the range of communication between the GCS and the UAV, as a result. The accuracy of this approach suffers significantly in the loss of GPS signal as well as when tracking system moves from outdoor to indoor environment where there is no clear LoS. Some researchers have proposed the use of anchor nodes equipped with passive radars to track UAV on the loss of GPS signals [109,110]. To address the issue of GPS positioning error, Allan variance (AVAR) has been proposed [111]. Notwithstanding, navigation errors can arise from poor satellite visibility or less than optimum geometry from the satellites that are visible. Accuracy of ephemeris data (i.e., each satellite’s positional information) is fundamental to the accuracy of the system. There are external effects that will affect the GPS signal which introduces errors and, subsequently, affect accuracy [54]. Multipath ranging errors can be caused by reflections of the GPS signals from mountains and tall buildings. In the event of poor satellite coverage for defined periods (typically less than 30 s), the system uses other navigation sensor inputs to enter into a dead reckoning mode. For prolonged periods of poor satellite reception, the system must reenter the acquisition mode.

#### 4.3.4. UAV Tracking Based on Monopulse

**Figure 12:**
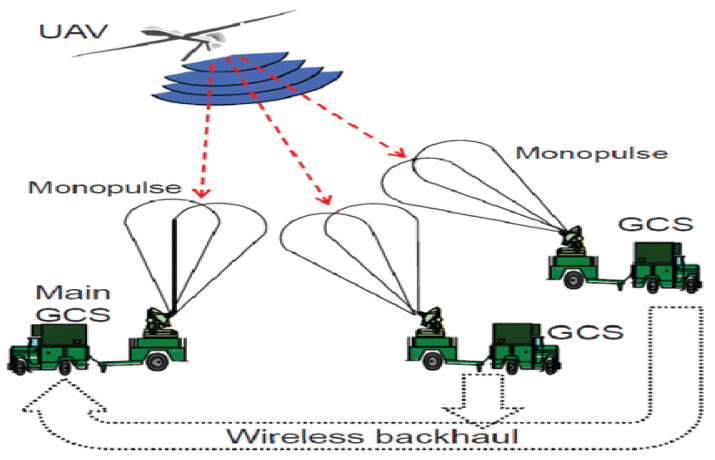
Monopulse UAV tracking [114].

In the absence of a GPS module, the most reliable approach to estimate the angle/direction of a received beam is via the monopulse technique. Monopulse is an advanced signal processing technique widely deployed in radar technology to resolve the angular position of targets with the aid of antenna arrays and beam-forming weights [112,113]. Monopulse relies on the extracting the sum and differences of the resulting overlapping beam patterns to obtain the angular position of the target. Hence, monopulse is a multi-beam system technology. In this fashion, the monopulse is able provide the five most fundamental UAV tracking parameters of: position vector, velocity vector, yaw angle, roll angle, and pitch angle. Unlike the previously listed tracking methods, UAV can be tracked using the beam formed by the directional antenna on the ground control station, as shown in Figure 12 [113]. Antennas create beam and, as such, the signal received from the UAV is stronger when the UAV is at the center of the beam. However, the signal becomes weaker when the UAV yaws or elevates, thereby moving away from the center of the beam of the ground station antenna that has been pointing at it. Thus, the movement of the UAV is detected but its direction is not known. This limitation can be addressed by the use of multiple GCS, as shown in Figure 12, in which all the GCS report their estimated UAV position to the main GCS which combines the angular estimates and decides the UAV site [114]. Motivated by the positive prospect of a monopulse UAV tracking technique, several works have been implemented in this regard.

Reference [115] proposed the use of phase calibration method for single-channel monopulse tracking (SCMT) systems based on error voltage’s convergence trajectory data facilitated by curve fitting techniques. By exploiting commercial off-the-shelf (COTS) equipment, a three-channel monopulse tracking has been designed [116]. Reference [117] presented a mathematical closed-form solution for identifying two targets based on a four-channel monopulse including both phase and amplitude monopulse measurement. There have been suggested strategies to localize multiple unresolved extended targets [118]. Apart from the major UAV antenna tracking schemes discussed so far, there are other strategies that has been proposed and highlighted in Table 2.

## 5. Multiple Input Multiple Output Technology

**Figure 13:**
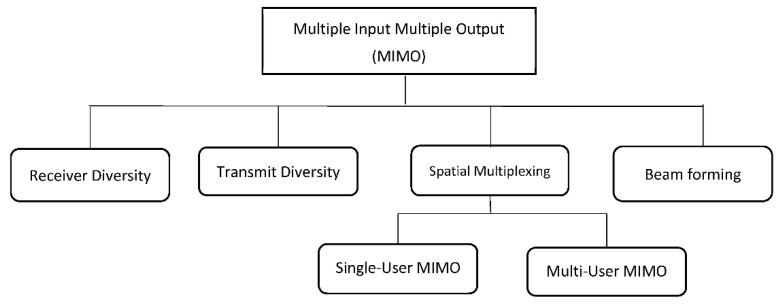
MIMO classification.

The multiple input multiple output (MIMO) technology is a promising technology capable of addressing the some of the conventional UAV tracking limitations in terms of design complexity, cost, energy efficiency, and deployment speed. Figure 13 shows the MIMO classification. In this section, we present an overview of MIMO classification and proposed a sectorized beam-forming technique as a scheme that can be adapted for UAV tracking antenna technology. The deployment of MIMO technology will only be maximized in a fading channel where there are channel multi-paths.

### 5.1. Receive Diversity

**Figure 14:**
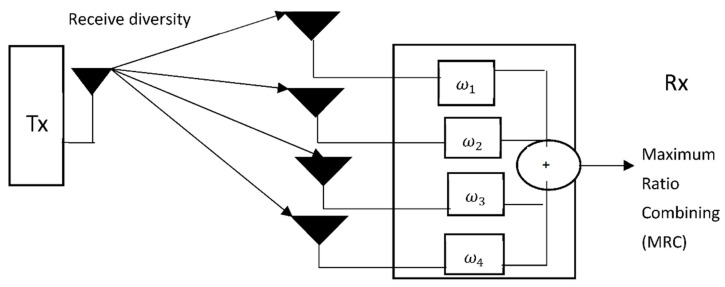
Receive diversity in MIMO.

Receive diversity is considered as one of the most widely deployed multi-antenna configurations in MIMO technology [132,133]. Receive diversity can come in different patterns of single input single output (SISO) with one receive antenna as well as a single input multiple output (SIMO) involving multiple receive antennas. A schematic diagram of receive diversity is shown in Figure 14. From the context of UAV deployment, the transmitter (Tx) will be the GCS, while the receiver (Rx) will be the aerial airborne platform. *ω* denotes the channel weights assigned to different antenna array in the MIMO antenna configuration. The different signals from multiple wireless links are combined via maximum ratio combining (MRC) [134,135] and selection combining (SC) [136,137]. The number of antenna array that can be fitted into the airborne platform depends on the transmitting radio frequency. The goal of receive diversity is more towards improving link reliability than increasing the signal rate. This scheme does not maximize the spectrum.

### 5.2. Transmit Diversity

Transmit diversity is the reverse of the receive diversity. In transmit diversity, the transmitter is equipped with multiple antennas at the transmitter to introduce diversity by transmitting redundant versions of the same signal. The Alamouti technique is a popular algorithm among the transmit diversity techniques [138]. There are various versions of transmit diversity, such as space-time block coding (STBC) and space-frequency block coding (SFBC). With STBC [139,140], signals are mapped as space and time to exploit the diversity present in the time domain. SFBC is operationally different from STBC because it exploits the antenna spacing and frequency domains encoding in the antenna [141,142]. Just like in receive diversity, transmit diversity does not increase UAV tracking antenna data rates. However, the scheme can enhance link reliability which is the primary goal of the UAV tracking antenna system. Both STBC and SFBC signals can be received using appropriate finite filter design.

### 5.3. Spatial Multiplexing

**Figure 15:**
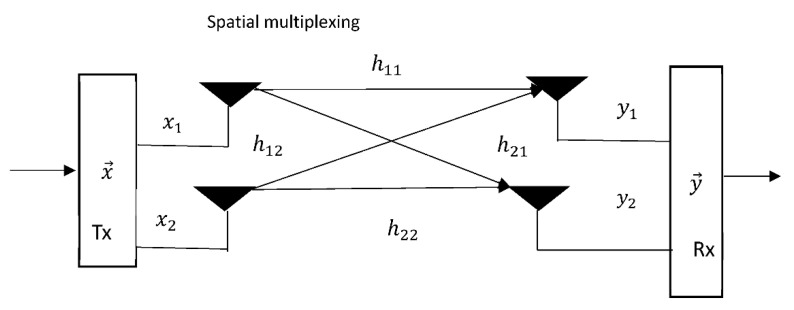
Schematic presentation of spatial MIMO.

Spatial multiplexing involves the transmission of independent parallel information streams to boast the data rate and enhance spectrum efficiency. Spatial multiplexing allows the system designer to use a different modulation code to modulate information bits and send them to various antenna ports [143,144]. The two most notable spatial multiplexing receiver algorithms are zero forcing (ZF) [145,146] and minimum mean-square error (MMSE) [147,148]. A schematic diagram of spatial multiplexing is depicted in Figure 15.

Spatial multiplexing in MIMO are of two variants namely, single-user MIMO (SU-MIMO) and multi-user MIMO (MU-MIMO). In SU-MIMO design configuration, all the elements of the signal vector $\vec{x}$ belong to a single user in this case, and perhaps for one UAV receiver array antenna. While, in MU-MIMO, the data streams of different users are multiplexed on different antenna and transmitted over the wireless channel coefficient h. In the context of UAV tracking antenna, this might involve transmitting the tracking parameters and information signals simultaneously.

### 5.4. MIMO Beam Forming

**Figure 16:**
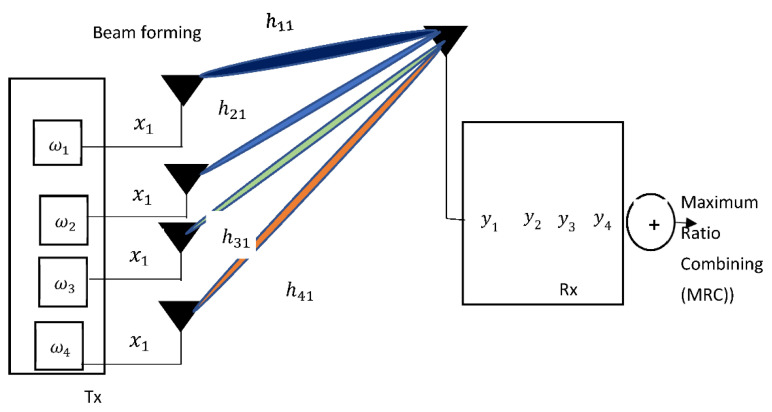
Schematic Diagram of Transmit MIMO beamforming.

MIMO beam-forming is an antenna technology in which multiple antenna arrays are deployed at the transmitter side to align the overall antenna radiation to the desired angle. The various antenna arrays produce the different signal beams which are responsible for the overall antenna gain (azimuth-wise) in the direction of the target herein the airborne platform. As could be in Figure 16, a transmit beam forming technique is the reverse of receive diversity. The depicted diagram could be construed as MISO because of the many input and single output. The signal at the receiver which is the airborne platform is stated as

(4) $$\vec{y}=\tilde{h}x+\tilde{n}$$

where $\vec{y}$ denotes the received signal vector, $\tilde{h}$ is the channel coefficient which can be modeled as Rician if there is clear LoS or Rayleigh if there are scatterers which will result in multi-paths, x is the information symbol that the UAV GCS intends to transmits to the airborne platform, and $\tilde{n}$ is the noise vector normally denoted as additive white Gaussian noise (AWGN). The sum of independent links present at the receiver is stated

(5) $$\tilde{y}=y_{1}+y_{2}+y_{3}+\cdots +y_{L}$$

$\left\{y_{1},\;\cdots y_{L}\right\}\;$ represent the various received independent complex wireless signals. Instead of making use of selection combining (SC), a maximum ratio-combining (MRC) algorithm will be used as it is more appropriate herein. By using the MRC algorithm, the aggregate sum of the links is stated as:

(6) $$\mathrm{MRC}=\omega_{1}^{*}y_{1}+\omega_{2}^{*}y_{2}+\omega_{3}^{*}y_{3}+\cdots +\omega_{L}^{*}y_{L}$$

$\omega_{1}^{*}$ connotes the beam weight complex transpose conjugate. The beam weight is synonymous to θ which is the desired angle of arrival (AoA) of the incoming signal of Equation (1). The AoA is the mechanical version of the beam weight complex which is the electronic version of AoA. Equation (6) leads to the derivation of the beam-forming stated as ${\bar{\omega }}^{H}\bar{y}$. Note that ${\bar{\omega }}^{H}$ is the Hermittian weight of the beamforming. ${\bar{\omega }}^{H}$ is also known as the beamformer. The beamformer output is mathematically derived as:

(7) $$\begin{array}{ll} Beamformer_{output\;} & ={\bar{\omega }}^{H}\left(\bar{h}x+\bar{n}\right)\\ & ={\bar{\omega }}^{H}\bar{h}x+{\bar{\omega }}^{H}\bar{n} \end{array}$$

From Equation (7), it could be seen that beamformer output consists of signal component ${\bar{\omega }}^{H}\bar{h}x$ and the noise component ${\bar{\omega }}^{H}\bar{n}$. The second line of from Equation (7) simply expanded the first line of the equation. By applying some advanced signal-processing techniques, in the absence of interference, the noise component reduces to:

(8) $${\bar{\omega }}^{H}\bar{n}=\sigma_{n}^{2}{\left\|w\right\|}^{2}$$

‖ ‖ is the norm of the vector and *σ*^2^ is the variance. It could be seen that the problem of beam-forming reduces to that of obtaining a close-form expression of the weight vector stated as

(9) $$\bar{\omega }=\bar{C}\bar{h}$$

where $\bar{C}$ is a constant. The optimal beamfomer is the antenna coefficient vector scaled by some constant shown in Equation (10)

(10) $$C^{2}{\left\|h\right\|}^{2}=1$$

$$C=\frac{1}{\left\|h\right\|}$$

Substituting Equation (9) into Equation (10) leads to the derivation of the optimal beamfoming vector that that maximizes the received the SNR as

(11) $${\bar{\omega }}_{opt}=\frac{\bar{h}}{\left\|h\right\|}\bar{C}\bar{h}$$

The problem of beam-forming simply reduces to computing the channel coefficient. The channel models must address the multipath scattering effects, time dispersion, and Doppler shifts that arise from relative motion between the transmitter and receiver. The beamformer can work with IMU sensors whose function is to feed the system with position of the airborne platform. Several techniques are available to decode the instantaneous channel coefficients, such as pilot signal, channel sounding, semi-blind estimation [149], blind estimates [150], and feedback channels [151,152].

### 5.5. Limitations of Deploying MIMO for UAV Tracking

MIMO beam-forming techniques can adequately replace the mechanical beam-steering technique of antenna UAV tracking. However, before the full adoption of the technique, the following issues affecting MIMO technique must be addressed.

***(a)***  ***Pilot Contamination:*** Pilot contamination is a scheme in MIMO technology in which the pilots or preambles are transmitted by the transmitter to acquire the channel state information for the determination of the beam weights [153,154,155]. The issue is very crucial and critical, and, as such, many researchers have proposed various strategies to address the issue. Reference [156] proposed the use of transmit power control schemes; reference [157] suggested the use of graph coloring scheme, in which difference nodes are assigned difference colors to mitigate against interference; and reference [158] studied the downlink capacity under massive MIMO pilot contamination.
***(b)***  ***MIMO Rank Deficiency:*** MIMO technology thrives under the assumption that the various multipath channels paths can be fully decoupled. Therefore, resulting to a full rank [159,160]. This implies that the channels are not carrying redundant information and instead of the signals to be uncorrelated, they are related. Rank deficiency is usually experienced in MIMO technology when there are insufficient scatterers in the environment which leads to modelling the channel as a Rician probability density function (PDF). This is instead of the popular Rayleigh fading channel with Rayleigh PDF with lots of delayed path. This issue can be addressed by introducing artificial scatteres in the signal path to induce NLoS scenario.
***(c)***  ***MIMO Complexity:*** The computational complexity of MIMO technology as we migrate from the domain “Non- Massive MIMO” to “ Massive MIMO” regimes. In massive MIMO, the antenna array cardinality increases in contrast to non-massive MIMO configuration. It is not surprising that massive MIMO can support numerous users in the context of MIMO multiuser (MIMO-MU). Using this technique, a GCS equipped with MIMO-MU can adequately support and control many UAVs, simultaneously. In other to address this issues, several MIMO complexity models have been proposed [161,162,163]. Most of the proposed strategies are centered on optimal antenna selection criteria.

### 5.6. Sectorized Beam-Forming Antenna for UAV Tracking

**Figure 17:**
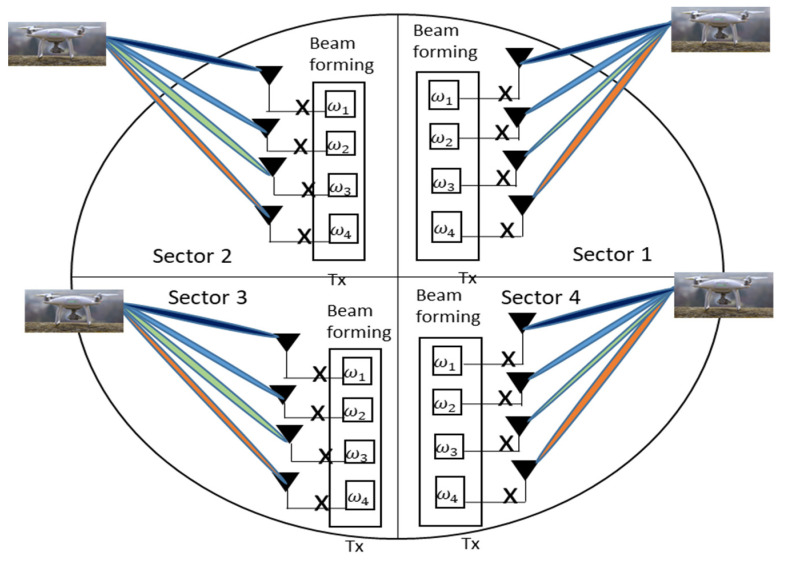
A four-sector beam-forming antenna for UAV tracking.

Sectorized antenna beamforming has the potential to be deployed for UAV tracking. Sectorized antenna configuration requires dividing the sphere into arbitrary sectors, such as 2, 3, 4, 6 sectors, as the case may be for effective tracking of the UAV. In our proposed solution, as shown in Figure 17, we depicted a four-quadrants uniform planar array (UPA) to track the UAV in all the sectors. The support for MIMO-BF lies in the fact that it can exploit the instantaneous channel attributed to align the beam weight and, hence, is reconfigurable. Secondly, a different modulation scheme could be adopted to respond to the varying channel state information, Thirdly, by introducing a delay circuit, it can alter the time and frequency components of the signal and hence, track the UAV dynamic flight parameters. By changing the antenna fed with the delay, the beam could rotate in four 90 degree steps. Beam-forming antenna can be etched using microstrip material because of the desirable form factor which can be easily fitted into the UAV GCS with easy. Furthermore, slot antenna configuration has interesting possibilities as well. The active sector can be electronically switched based on the RSSI or the beam weights which are computed by the GCS module. From the context of antenna polarization, circular polarization will be ideal in this fashion when compared to horizontal and vertical polarization. Circular polarization will ensure that there is no polarization mismatch, which will be result in the loss of signal.

### 5.7. Future Research Trends

A UAV antenna tracking is an emerging technology with great potential to address the needs of hobbyist and professionals. As the technology gains extensive visibility, it has become necessary to conduct further studies on the following.

***i*.**  ***Energy Efficiency:*** Generally, UAVs suffer from the problem of reduced size, weight, and power (SWaP). Energy efficiency is crucial to UAV antenna tracking, especially when mechanical beam steering tracking is involved [164,165]. Energy efficiency will also become critical if MU-MIMO is adopted as the core of UAV antenna tracking. Hence, more system modelling and energy efficiency is needed.
***ii*.**  ***Wireless Channel Model:*** There is a need for adequate UAV wireless channel modelling, considering the fact that UAV is generally regarded as a LoS communication system. This assumption might not be feasible considering that there are lots UAV hobbist in crowded urban areas [166,167]. Secondly, the transmission range of UAVs varies according to the equipped transceiver pairs. It is plausible that the dynamic channel model will be develop to cater for this variation.
***iii*.**  ***Performance of UAV Tracking Antenna in Poor Channel:*** Studies on the performance of UAV tracking antenna in poor channels are yet to be fully comprehended [168]. Most significantly, this study is imperative to avoid crashing the UAVs when the UAV is in critical mission. Therefore, a model must be developed to analyze this performance, in the absence of first person view (FPV) module.
***iv*.**  ***Machine Learning:*** Machine learning is an evolving technology that can enhance the UAV tracking antenna system in terms of reduction in beam mis-alignment and bean weight prediction. Machine learning algorithms are divided into supervised learning, unsupervised learning (classification), and reinforced learning [169,170]. Towards this trend, machine learning beam tracking and weight optimization for mmwave multi-UAV links has been proposed [171].
***v*.**  ***Steering Beam Alignment Error:*** Unlike other mobile nodes, UAV tracking must address the issue of beam alignment resulting from angular Doppler as the UAV moves from one place to the next. Steering beam alignment is not only used for the mechanical beam steering approach. This can also be experienced by the electronic beam steering resulting from poor channel modelling and incomplete channel state information. Hence, this opens up a great research area.

## 6. Conclusions

The UAV antenna tracking is an important module in the UAV GCS unit because it is responsible for aligning the radio control (RC) signals from the UAV GCS to the airborne platform. Without proper tracking mechanism, there will be a loss of many airborne platforms. In this work, we have extensively reviewed all the currently deployed UAV antenna tracking systems and proposed the use of sectorized beam-forming for a UAV antenna. The choice of any UAV antenna tracking must be analyzed from the perspective of low cost, energy consumption, memory size, and low processing capabilities.

## Figures and Tables

**Table 1:** Comparing Antenna type gain and beamwidth relationship [54].

Antenna Type	Gain (dBi)	Beamwidth (Degrees)
Vertical half-wave dipole	0	360
Vertical quarter-wave with ground plane	0	360
Four-element Yagi	6	43
UHF corner reflector	9	27
Two stacked vertical half- wave dipoles	3	360
Small horn antenna for use at 10 GHz	10	20
3 m diameter parabolic antenna for tracking space vehicles at UHF	40	4

**Table 2:** The other strategies that have been proposed.

Reference	Observation
[119]	Conducted optimal numerical analysis of electronically-steered arrays onboard electrically-large platforms based on FEKO software.
[120]	Implemented an analog beam tracking utilizing Cramer–Rao lower bound algorithm that enables high tracking speed and data rate for UAV.
[121]	Proposed a 7-dBi-high gain steering beam consisting of three circular array of two Yagi–Uda antennas resulting in seven beams which cover the 0–180-degree azimuth plane.
[122]	Designed array antenna at the receiver side to detect the Angle of Arrival (AoA) of the target.
[123]	Deployed frequency difference of arrival (FDOA) for UAV localization which employs several weighted least-squares minimizations only and does not require initial solution guesses to obtain a location estimate.
[124]	Proposed an architecture consisting of attitude heading and reference system (AHRS) for marine satellite tracking antennas (MSTAs) to overcome attitude disturbance due to ship vibration and rotation motion.
[125]	Fault tolerant control (FTC) system was used for the satellite tracking antenna which aligns the onboard antenna toward a chosen satellite while the high sea waves disturb the antenna.
[126]	MATLAB-based tracking system simulator was also developed to test the control system performance of a rooftop antenna tracking system.
[127]	Leveraged on the desirable attributes of slotted wave guide array (SWGA) technology, an innovative dual polarization antenna working at Ku band (14~14.5 GHz) was fabricated.
[128]	Design, fabrication, and testing of a helical antenna using 3D printing technology operating at 5 GHz. Several commercially-available dielectric printers and materials (e.g., PLA, ABS, PC) were evaluated.
[129]	Achieved a blind beam tracking for Ka-band UAV satellite communication system, where UAV is equipped with a large-scale antenna array.
[130]	A priori knowledge aided channel tracking method was proposed in for Teraherz massive MIMO system, where a linear motion is adopted to derive angles of the incident signals and then the complex gains are estimated by pilots.
[131]	Presented a scheme referred as an angle division multiple access (ADMA) based channel tracking scheme was proposed in for massive MIMO systems.

## Data Availability

Not applicable.
